# Six to 12-year outcomes of magnetic sphincter augmentation for gastroesophageal reflux disease

**DOI:** 10.1038/s41598-020-70742-3

**Published:** 2020-08-13

**Authors:** Davide Ferrari, Emanuele Asti, Veronica Lazzari, Stefano Siboni, Daniele Bernardi, Luigi Bonavina

**Affiliations:** 1 Department of Biomedical Sciences for Health, University of Milan, Milan, Italy; 2 Division of General and Foregut Surgery, IRCCS Policlinico San Donato, Piazza E. Malan, 1, 20097 San Donato Milanese, MI Italy

**Keywords:** Gastrointestinal diseases, Outcomes research

## Abstract

The magnetic sphincter augmentation (MSA) device has been proven safe and effective in controlling typical reflux symptoms and esophageal acid exposure for up to 6-year follow-up. Longer term outcomes have not been reported yet. A prospectively maintained database was reviewed to assess long-term safety and efficacy of the laparoscopic MSA procedure at a single referral center. Gastro-Esophageal Reflux Disease-Health Related Quality of Life (GERD-HRQL), use of proton-pump inhibitors (PPI), and esophageal acid exposure were compared to baseline. Favorable outcomes were defined as ≥ 50% improvement of GERD-HRQL total score and PPI discontinuation. Between March 2007 and March 2020, 335 patients met the study inclusion criteria, and 124 of them were followed from 6 to 12 years after surgery (median 9 years, IQR 2). Mean total GERD-HRQL score significantly improved from 19.9 to 4.01 (*p* < 0.001), and PPI were discontinued by 79% of patients. The mean total percent time with pH < 4 decreased from 9.6% at baseline to 4.1% (*p* < 0.001), with 89% of patients achieving pH normalization. Independent predictors of a favorable outcome were age at intervention < 40 years (OR 4.17) and GERD-HRQL score > 15 (OR 4.09). We confirm long-term safety and efficacy of MSA in terms of symptom improvement, decreased drug dependency, and reduced esophageal acid exposure.

## Introduction

The global burden of gastroesophageal reflux disease (GERD) is enormous, with a pooled prevalence of 13.3% in community-based studies^1^. Symptoms and complications of GERD persist in up to 40% of patients treated with proton-pump inhibitors (PPI)^2,3^, and fundoplication is largely underused because of the steep learning curve and reported variability in outcomes^4^. The aim of fundoplication is to restore lower esophageal sphincter (LES) function by remodeling the esophagogastric junction^5^. Both total (Nissen) and partial (Toupet) fundoplication procedures require mobilization of the gastric fundus to be wrapped around the distal esophagus^6^. To further enhance the antireflux barrier, a crural diaphragmatic repair is routinely added^7^. Currently, the fact that fundoplication is offered to less than 1% of the GERD population may have an impact on the progression of symptoms and the development of Barrett’s esophagus^8^.

The magnetic sphincter augmentation (MSA) procedure (Linx Reflux Management System, Ethicon, Johnson & Johnson, Shoreview, Mn, USA) was developed as a less disruptive and more standardized and reproducible laparoscopic surgical option for the treatment of GERD. The MSA device is composed by a variable number of interlinked titanium beads with a magnetic core inside. This ring-like system produces a magnetic force that augments the LES. The first feasibility trial and a large prospective nonrandomized study were published in 2008^9^ and 2013^10^, respectively. The MSA procedure has been granted approval for clinical use by the Food and Drug Administration in 2012. Previous reports from our group have shown feasibility, safety, and efficacy of the MSA procedure up to 6 years of follow-up^11,12^. We now provide the long-term outcomes of a cohort of patients followed for a minimum of 6 years.

## Subjects and methods

The study was a single-center, retrospective, single-arm study, where patients served as their own controls. The study protocol was approved by the Internal Review Board of IRCCS Policlinico San Donato (HSD 2019-072), and the research was performed in accordance with the relevant regulations. Informed consent was obtained from all study participants. The prospectively maintained database was reviewed to assess long-term safety and efficacy of the MSA. All patients who underwent a MSA procedure between March 2007 and March 30, 2020 were included in the study. Data analysis was performed in the whole group of patients and in a cohort of individuals followed for 6- to 12 years. Gastro-Esophageal Reflux Disease-Health Related Quality of Life (GERD-HRQL), use of proton-pump inhibitors (PPI), and esophageal pH monitoring parameters were compared to patients’ own preoperative data. Favorable outcome of the MSA procedure was defined as ≥ 50% improvement in GERD-HRQL total score and PPI discontinuation.

### Preoperative assessment and patient selection

Before surgery, all patients referred for surgical therapy of GERD were evaluated by a multidisciplinary team including gastroenterologists, dieticians, and clinical psycologists at our center. The diagnostic assessment included the foregut symptom questionnaire and the GERD-HRQL questionnaire, upper gastrointestinal endoscopy, barium swallow study, ambulatory esophageal pH monitoring, and esophageal manometry. The foregut symptom questionnaire gives a score for heartburn, regurgitation, dysphagia, and chest pain on a scale of 0 to 4 (grade 0, none; grade 1, less than once a week; grade 2, several times a week; grade 3, daily, affecting lifestyle; grade 4, always, markedly affecting lifestyle. The GERD-HRQL score consists of 10 questions that specifically address GERD symptoms. Each question has a score ranging from 0 to 5, and the total score ranges from 0 to 50^13^. Preoperative determination of hernia size was based on upper gastrointestinal endoscopy and barium swallow study. Endoscopy evaluated the presence of hiatus hernia, morphology of the gastroesophageal valve using the Hill classification, grade of esophagitis using the Los Angeles classification, and the presence of Barrett esophagus using the Prague classification. Ambulatory esophageal pH monitoring was performed using trans-nasal pH-impedance equipment or the Bravo wireless system (48–96 h pH study). Measurements collected from esophageal pH testing included the DeMeester score and its individual components. A standard or, more recently, a high-resolution manometry were performed^14^. Main parameters investigated were the resting pressure and length of the LES, the distal esophageal amplitude (DEA) and/or the distal contractile integral (DCI), and the percent of effective contractions. An esophageal amplitude of less than 40 mm Hg and a greater than 50% of non-transmitted swallows indicated ineffective esophageal motility. Initial criteria for patient selection were the following: persistent reflux symptoms despite optimal PPI therapy, abnormal esophageal acid exposure confirmed by ambulatory esophageal pH monitoring, hiatus hernia < 3 cm, esophagitis < grade B, body mass index < 35 kg/m^2^, and absence of specific motility disorders. With further clinical experience and research, the criteria have been expanded to include patients with larger hiatus hernia, short Barrett’s esophagus, and mild esophageal dysmotility. The MSA procedure was not offered to patients with recurrent GERD after failed fundoplication or other surgical/endoscopic procedures at the esophagogastric junction, and to those with known history of nickel allergy or eating disorders.

### Surgical approach

The MSA device was implanted via laparoscopy as previously described^15^. Under general anesthesia, the esophago-gastric junction was exposed following incision of the peritoneal reflection. The posterior vagus nerve was identified and separated from the esophagus for a length of about 1 cm. No short gastric vessels were divided. The esophageal circumference was measured with an appropriate magnetic sizer inserted through the retroesophageal tunnel. A minimal or formal posterior crura repair was performed depending on the size of the hiatal defect and the degree of hiatus hernia. Over the study period and starting from 2014, modifications of the surgical technique occurred. First, formal mediastinal dissection became routine practice; second, a new generation MSA device was introduced for use in magnetic resonance up to 1.5 T; and, third, a new generation sizer device for measuring the esophageal circumference was introduced.

### Postoperative assessment and follow-up

All patients underwent a comprehensive clinical evaluation including the foregut symptom and the GERD-HRQL questionnaires, use and dosage of PPI, esophageal pH measurements, upper gastrointestinal endoscopy, and esophageal manometry. The treatment was considered successful if, compared to baseline, at least a 50% reduction in the total GERD-HRQL score and PPI discontinuation or at least a 50% dose reduction was achieved.

### Statistical analysis

Continuous data are reported as median ± interquartile range (IQR) or mean ± standard deviation (SD). Patients served as their own control, and pre- and postoperative data were compared using the two-tailed, paired Student’s t test. Categorical demographic and baseline variables are reported as proportions or frequencies, and compared using Wilcoxon test for continuous outcomes and McNemar’s test for paired samples. A *p* value < 0.05 was considered statistically significant. Parameters of the univariate analysis with *p* < 0.05 were included in a multivariate logistic regression test to determine what independent variables might predict the clinical success of the surgical procedure. A *p* value < 0.05 was considered statistically significant. Statistical analyses were performed using SPSS software 23.0 (IBM, Armonk, New York, U.S.).

## Results

**Figure 1:**
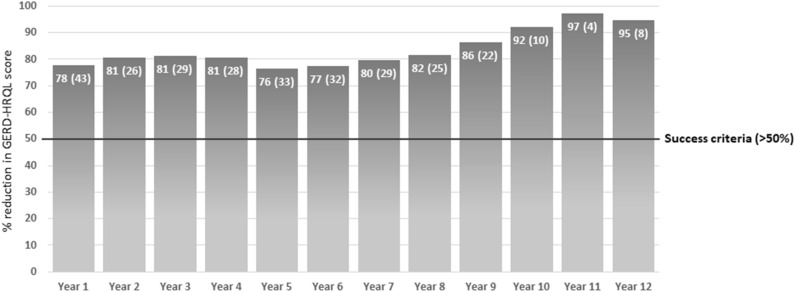
Average percent reduction (± SD) of total GERD-HRQL score per year over the follow-up.

Between March 2007 and March 30, 2020, a total of 1,052 patients underwent laparoscopic surgery for GERD at our institution. A Toupet fundoplication was performed in 499 patients, Nissen fundoplication in 218, and MSA procedure in 335. The baseline demographic and clinical characteristics of patients who received MSA are listed in Table 1. Two patients died during the follow-up for unrelated reasons. Overall, there was more than 50% reduction in the total GERD-HRQL score compared to baseline in each year of follow-up (Fig. 1). Table 2 shows the median GERD-HRQL scores by question.

**Table 1:** Baseline patient characteristics (continuous variables expressed as median (IQR)). *BMI = Body Mass Index; ^†^PPI = Proton Pump Inhibitors; ^§^GERD-HRQL = Gastro-Esophageal Reflux Disease Health Related Quality of Life; ^‡^LESP = Lower Esophageal Sphincter Pressure; ^※^DEA = Distal Esophageal Amplitude; ^δ^IEM = Ineffective esophageal motility.

	F.U. < 6 years (n = 211)	F.U. 6–12 years (n = 124)
Age, years	46 (20)	44 (20.8)
Male, n (%)	139 (65.8)	83 (66.9)
BMI*, kg/m^2^	25.4 (5)	23.9 (4.5)
Duration of symptoms, years	8 (11.3)	6 (7)
PPI^†^ use, years	7 (7)	4 (6)
GERD-HRQL§ total score	19.5 (10)	21 (9.5)
**Esophagitis, n (%)**		
None	167 (79.1)	103 (83.1)
Grade A	22 (10.4)	11 (8.9)
Grade B	18 (8.5)	9 (7.2)
Grade C	2 (1.0)	1 (0.8)
Grade D	2 (1.0)	0 (0.0)
Barrett’s esophagus, n (%)	10 (4.7)	4 (3.2)
**Hiatal hernia length, n (%)**		
None	57 (27.0)	18 (14.5)
1 cm	24 (11.4)	37 (29.8)
2 cm	70 (33.2)	44 (35.6)
3 cm	35 (16.6)	20 (16.1)
≥ 4 cm	25 (11.8)	5 (4.0)
Basal LESP^‡^, mmHg	14.2 (15.4)	15.1 (12)
DEA※, mmHg	66 (40)	63.0 (34.2)
IEM^δ^, n (%)	19 (9.0)	1 (0.8)
DeMeester score	24.8 (26.8)	31.3 (24.6)
% total time pH < 4	6.4 (6.8)	8 (6.6)

**Table 2:** Summary of median (IQR) GERD-HRQL scores by question. *GERD-HRQL = Gastro-Esophageal Reflux Disease Health Related Quality of Life.

	Baseline	< 6 years	6–12 years
	n = 124	n = 211	n = 124
How bad is your heartburn?	4 (2)	2 (2)	2 (2)
Heartburn when lying down?	4 (2)	0 (0)	0 (0)
Heartburn when standing up?	3 (2)	0 (0)	0 (0)
Heartburn after meals?	4 (2)	1 (2)	0 (2)
Does heartburn change your diet?	2 (2)	0 (1)	0 (0)
Does heartburn wake you from sleep?	2 (3)	0 (0)	0 (0)
Do you have difficulty swallowing?	0 (0)	0 (0)	0 (0)
Do you have bloating and gassy feelings?	0 (2)	1 (2)	0 (1.5)
Do you have pain with swallowing?	0 (0)	0 (0)	0 (0)
If you take medication, does this affect daily life?	0 (1)	0 (0)	0 (0)
Total median GERD-HRQL* score	21 (9.5)	4 (5)	3 (5.5)

### Postoperative adverse events and long-term safety profile

Adverse events were assessed from the time of implant through to the final visit. The rate of procedure-related adverse events was 11.6% (39/335) throughout the overall study period.

Eight patients (2.4%) required a single endoscopic pneumatic dilation due to persistent dysphagia at 11, 13, 21, 23, 28, 53, 60, and 65 months, respectively, after surgery. Thirty-one patients (9.2%) required laparoscopic device removal for various reasons (Table 3). The most common one-stage remedial procedure was a laparoscopic Toupet fundoplication (n = 18).

**Table 3:** Main reasons for magnetic sphincter augmentation device removal.

	< 6 years (n = 28)	6–12 years (n = 3)
Erosion	6	0
Regurgitation	6	0
Heartburn	5	1
Dysphagia	5	1
“Foreign body” sensation	2	0
Odynophagia	1	0
Pharyngodinia	1	0
Chronic cough	1	0
Need of magnetic resonance study	1	1

### Long-term (6–12 year) outcomes

One-hundred-twenty-four patients, who were implanted between March 2007 and February 2014, had a minimum follow-up of 6 years. The median follow-up was 9 years (IQR 2). At the latest follow-up, 92 of 124 patients (74.2%) did not report any esophageal symptom (grade 0–1 for heartburn, regurgitation, dysphagia, and chest pain). The mean total GERD-HRQL score decreased from 19.9 at baseline to 4.01 at the latest follow-up (*p* < 0.001); 89% of patients met the criteria of favorable long-term outcome, Clinically significant improvement in GERD-HRQL is also reflected by the reported patient satisfaction, which was achieved in 92.7% of patients. The prevalence of grade 2–4 regurgitation significantly decreased from 59.6% at baseline to 9.6% postoperatively (*p* < 0.01). At the latest follow-up, complete or at least 50% reduction in the average daily dose of PPI was achieved by 79% and 89.5% of patients, respectively.

**Figure 2:**
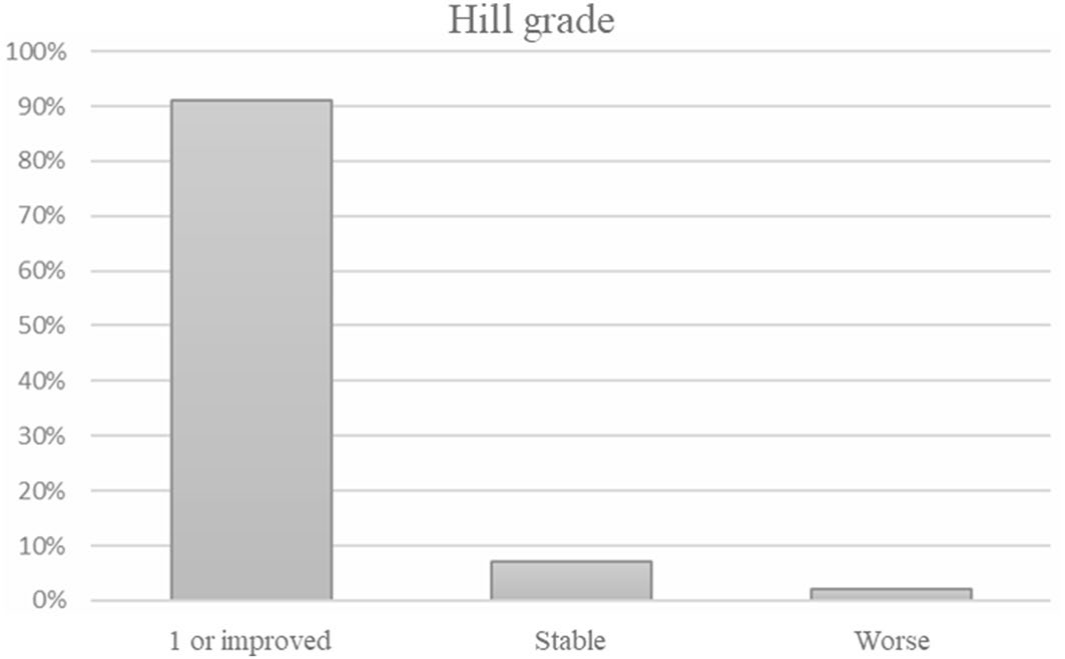
Changes of Hill grade classification in 45 patients after magnetic sphincter augmentation.

The majority of patients (86.3%) underwent upper gastrointestinal endoscopy after 6 years of follow-up. Hiatus hernia was found in 7 patients (6.5%), grade A esophagitis in 5 patients (4.7%), and incomplete intestinal metaplasia in 3 (2.8%). Four additional patients, who had been treated with radiofrequency ablation for short-segment Barrett’s esophagus without dysplasia before the MSA procedure, were followed endoscopically for up to 8 years without recurrence of intestinal metaplasia. The Hill grade was measured in 45 patients before and after surgery. At the latest endoscopic follow-up, 41 patients (91%) retained their preoperative Hill grade I or improved, 3 (7%) remained stable, and in 1 (2%) patient the Hill grade worsened (*p* < 0.01) (Fig. 2).

**Figure 3:**
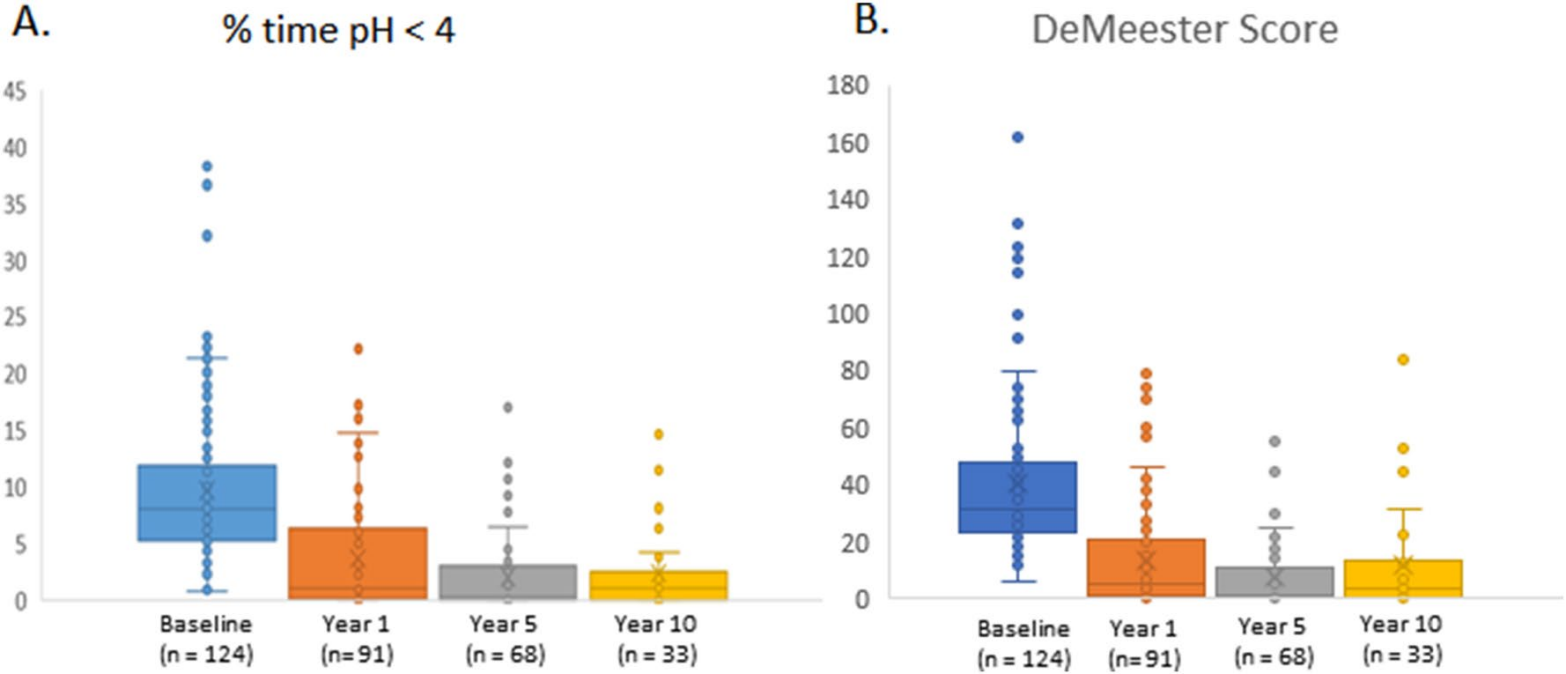
Median % time at pH < 4 (**A**) and median DeMeester score (**B**) in sequential pH studies compared to baseline.

Esophageal pH testing off PPI therapy showed that the mean percentage of time that pH was < 4 decreased from 9.7% at baseline to 4.2% at latest follow-up (*p* < 0.001). All the other pH monitoring variables were significantly reduced at 6–12 years compared with baseline (Table 4). Eighty-nine percent of patients who completed esophageal pH monitoring at 6- to 12 years follow-up achieved either normal esophageal acid exposure or had at least a 50% reduction compared to baseline. Sequential pH studies were performed in 37 patients at various time intervals since surgery (Fig. 3).

**Table 4:** Esophageal pH measurements (mean ± SD) off proton-pump inhibitors.

Measure	Baseline	6–12 years	*p*
	n = 124	n = 91	
**Total time (%)**			
pH < 4	9.7 (6.4)	4.2 (4.9)	< 0.001
Upright	9.7 (7.8)	4.6 (4.9)	< 0.001
Supine	8.3 (9.6)	3.3 (7.4)	< 0.001
**Reflux episodes**			
Total number	92.2 (92.2)	71.5 (67.7)	0.125
Number lasting > 5 min	6.1 (6.0)	4.3 (5.8)	0.036
Longest (minutes)	32.9 (34.2)	19.6 (31.5)	0.005
DeMeester score	40.7 (26.5)	16.3 (18.8)	< 0.001

Table 5 shows the long-term results in a subgroup of patients with follow-up longer than 10 years. Overall patient satisfaction, based on the question “would you undergo the operation again or recommend it to a friend?”, was 93.8%.

**Table 5:** Long term results in 32 patients with follow-up > 10 years. *GERD-HRQL = Gastro-Esophageal Reflux Disease Health Related Quality of Life.

	n = 32
Median GERD-HRQL* score	2
Dysphagia	0
Ability to belch	32 (100)
Ability to vomit	29 (90.6)
Occasional PPI use	7 (21.8)
Daily PPI use	3 (9.4)
Overall patient satisfaction	30 (93.8)

### Predictors of long-term clinical success

Eighty-one percent of patients had a successful clinical outcome, defined as GERD-HRQL score improvement > 50% and complete discontinuation of PPI use. At univariate analysis, age at intervention < 40 years, preoperative GERD-HRQL total score > 15, duration of symptoms, regurgitation, atypical symptoms and absence of generalized anxiety disorder were statistically significant as independent predictors of clinical success and were included in the multivariate logistic regression test (Table 6). At multivariate analysis, independent predictive variables of successful outcome were confirmed to be age < 40 years and GERD-HRQL score > 15 (Table 7).

**Table 6:** Potential predictors of success at univariate logistic regression model. *BMI = Body Mass Index; ^§^GERD-HRQL = Gastro-Esophageal Reflux Disease Health Related Quality of Life); ^‡^MSA = Magnetic Sphincter Augmentation.

Variable	*p*
Age at intervention (< 40 y)	**0.036**
Sex, male	0.975
BMI* (< 25)	0.361
Disease duration	**0.036**
GERD-HRQL§ total score (> 15)	**0.013**
General anxiety disorder	**0.043**
Typical symptoms	0.353
Atypical symptoms	**0.019**
Regurgitation	**0.021**
Esophagitis	0.936
Hiatal hernia	0.054
Barrett’s esophagus	0.489
LES basal pressure	0.600
LES overall length	0.442
Distal esophageal amplitude (< 43 mmHg)	0.468
DeMeester score	0.161
% total time pH < 4	0.409
Number of MSA^‡^ beads (> 14)	0.328

Statistically significant values/differences are indicated in bold.

**Table 7:** Independent predictive variables of success at multivariate analysis. **OR,** odds ratio; **B,** logistic regression coefficient; **SE,** standard error. ^□^GERD-HRQL = Gastro-Esophageal Reflux Disease-Health Related Quality of Life.

Variable	OR (CI 95%)	B	SE	*p*
Age at intervention (< 40 years)	4.61 (1.29–16.45)	1.527	0.649	**0.019**
GERD-HRQL^□^ total score (> 15)	4.19 (1.39–12.63)	1.432	0.563	**0.011**
Duration of disease	0.97 (0.89–1.06)	− 0.027	0.043	0.531
General Anxiety Disorder	0.35 (0.11–1.14)	− 1.042	0.599	0.082
Atypical symptoms	2.86 (0.99–8.23)	1.051	0.539	0.051
Regurgitation	1.88 (0.68–5.19)	0.630	0.519	0.225

Statistically significant values/differences are indicated in bold.

## Discussion

This 6- to 12-year follow-up study of a cohort of patients undergoing MSA confirms satisfactory and durable clinical outcomes over a median follow-up of 9 years. The present report corroborates the findings of two previous studies with up to 6-year follow-up documenting symptom relief, discontinuation of PPI, minimal side effects, and long-term safety^11,12^.

**Figure 4:**
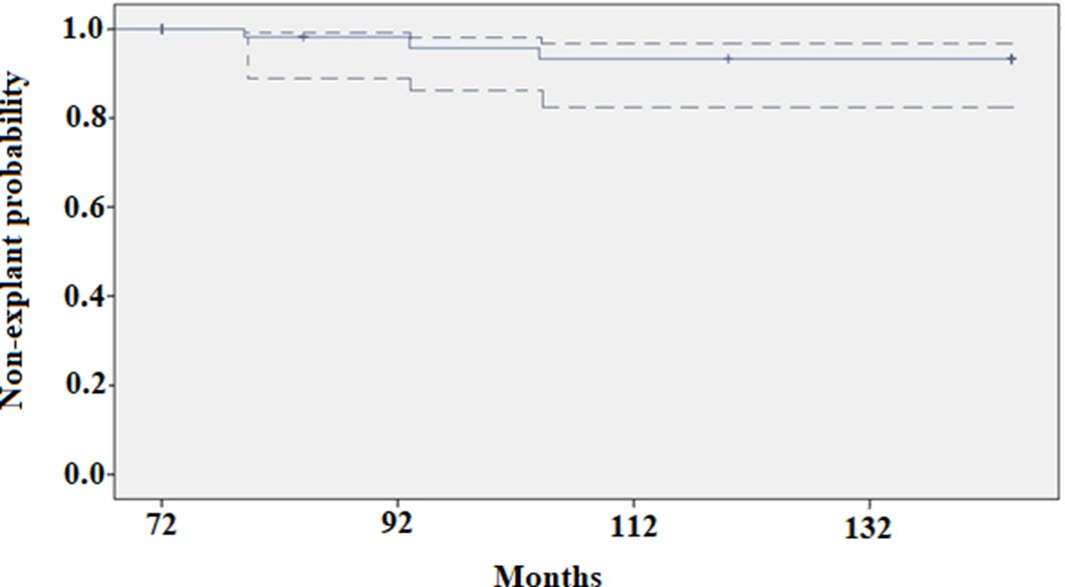
Kaplan–Meier estimate of explant-free probability over 6- to 12-year follow-up after magnetic sphincter augmentation (95% confidence interval indicated pointwise). [Graph created using SPSS software 23.0, URL = https://www.ibm.com/software/analytics/spss/register/].

The incidence of adverse events was low during the study time-frame, providing reasonable assurance that the risk of MSA complications does not increase with longer implant duration. The reasons for late device removal included dysphagia, continued reflux symptoms, and planned magnetic resonance imaging, but no erosions or migrations were observed. The overall reported rate of MSA device erosion is less than 0.5%; most events occurred within 4 years of the implant and have been managed electively without complications^16–18^. In our whole series, including 335 implants, most complications requiring MSA removal have occurred in patients implanted with a smaller (no.12 and 13) device. It should be noted that, during the study period, the sizer instrument has been replaced with a more user-friendly device in an attempt to improve the reproducibility of measurements. Furthermore, it has become clear over time that it is wiser to oversize by increasing 3 beads from the point of sizer release, and to use a larger MSA device to minimize dysphagia and decrease the likelihood of removal^19,20^. In our cohort of patients followed for 6–12 years, the overall estimated probability of MSA explant was 0.1 (Fig. 4).

**Figure 5:**
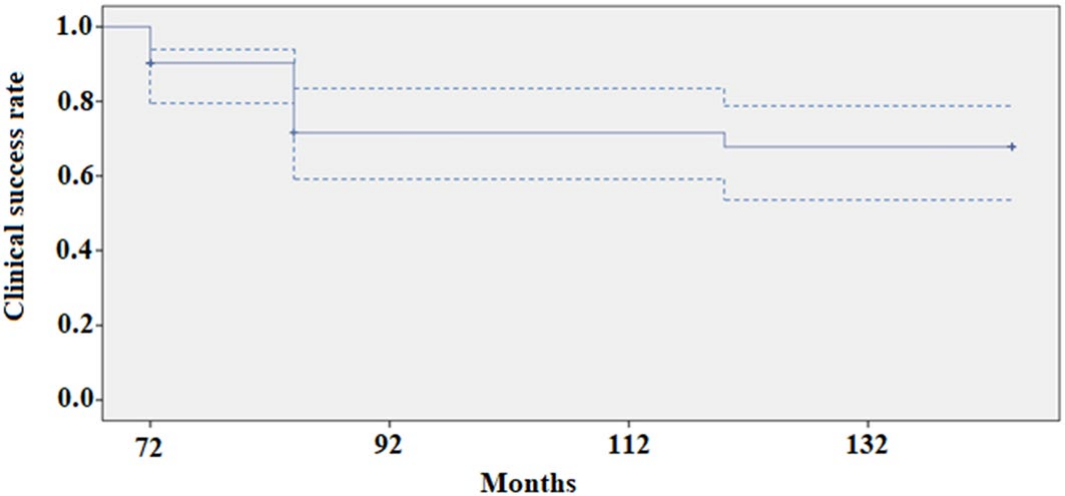
Kaplan–Meier estimate of clinical success (GERD-HRQL < 50% reduction or PPI discontinuation) over 6- to 12-year follow-up after magnetic sphincter augmentation (95% confidence interval indicated pointwise).[Graph created using SPSS software 23.0, URL = https://www.ibm.com/software/analytics/spss/register/].

The results of the present study show a 0.7 estimated probability of clinical success of at 6 to 12 years of follow-up (Fig. 5). The overall satisfaction rate of our patients was 92.5%. The prevalence of grade 2–4 regurgitation was significantly decreased (*p* < 0.01), and this is consistent with the one-year results of a recent randomized trial comparing the effect of MSA versus PPI^21^. Our multivariate analysis indicated that age < 40 years is an independent predictive variable of successful outcome. This is consistent with the study by Ayazi et al.^22^ who found that male sex was also an independent predictive factor. The fact that MSA is more effective in younger and male patients is of particular interest because this operation may have a profound impact on the course of GERD if performed in an earlier disease stage^23^. In addition, a recent population-based study showed lower recurrence rates after fundoplication in young men who would otherwise require several decades of PPI therapy^24^.

Although MSA was not directly compared with fundoplication in the present study, historical data from other clinical studies provide evidence that side effects typically associated with Nissen fundoplication, such as persistent dysphagia, gas bloat, and inability to belch/vomit, are less frequent and severe after the MSA procedure^25,26^. Of interest, disease-specific quality of life was similar in a propensity-score matched analysis comparing MSA and Toupet fundoplication^27^. In the present study, gas bloat and inability to belch/vomit were reported by 4% and 1.6% of patients, respectively.

The present study also suggests that the effect of MSA on esophageal acid exposure is sustained over time, as demonstrated by sequential pH studies showing reflux control up to 12 years of follow-up. Further, in a recent study, we showed that ineffective esophageal motility detected by high-resolution manometry can reverse to normal at a median of 12 months after MSA, and the only factor significantly associated to postoperative dysphagia was the presence of preoperative dysphagia^14^.

In recent years, it has become evident that the effectiveness of MSA can be enhanced by adding a formal crural repair^28,29^. The rationale behind this concept is that the extent of hiatus hernia can be underestimated both pre- and intraoperatively. Therefore, minimizing the amount of dissection performed and preserving the phreno-esophageal ligament may cause placement of the device below the true esophagogastric junction and may result in less effective reflux control. On the other hand, the MSA procedure is feasible even in large hernias based on our own clinical experience and other recent reports^30–34^.

Finally, encouraging data support the hypothesis that intestinal metaplasia can reverse after MSA^35^, especially in patients with short Barrett’s segments and in those with normalized DeMeester score. Although experience with MSA before or after radiofrequency ablation for Barrett’s esophagus is still very limited, we have successfully treated 4 patients who have been followed by endoscopy up to 8 years without recurrence.

The main limitations of this study are the retrospective design, the fact that there was no comparison treatment group, and the possible selection bias. However, despite the fact that criteria for patient selection, surgical technique, and type of device and sizer have evolved during the study period, the patient population of this study was homogeneous and indications for surgical therapy were consistent based on the preoperative pH study confirming GERD. Finally, this is the first report of a cohort of patients who completed the 6–12 year follow-up after MSA.

## Conclusions

When offered as a first-line surgical option, MSA allows durable control of symptoms and esophageal acid exposure, and improves patient quality of life up to 12 years of follow-up without significant safety issues. A preoperative GERD-HRQL total score > 15 and age below 40 years are independent predictive factors of favorable outcome. Based on the above results and the high levels of patient satisfaction, MSA may represent a true paradigm shift that has the potential to fill the current therapy gap in GERD. A randomized clinical trial comparing MSA and either total or partial fundoplication could provide more robust and definitive conclusions.
